# Synaptic circuits involving gastrin-releasing peptide receptor-expressing neurons in the dorsal horn of the mouse spinal cord

**DOI:** 10.3389/fnmol.2023.1294994

**Published:** 2023-12-07

**Authors:** Raphaëlle Quillet, Maria Gutierrez-Mecinas, Erika Polgár, Allen C. Dickie, Kieran A. Boyle, Masahiko Watanabe, Andrew J. Todd

**Affiliations:** ^1^School of Psychology and Neuroscience, University of Glasgow, Glasgow, United Kingdom; ^2^Department of Anatomy, Hokkaido University Graduate School of Medicine, Sapporo, Japan

**Keywords:** GRPR, anterolateral system, pain, itch, PSD95

## Abstract

The superficial dorsal horn (SDH) of the spinal cord contains a diverse array of neurons. The vast majority of these are interneurons, most of which are glutamatergic. These can be assigned to several populations, one of which is defined by expression of gastrin-releasing peptide receptor (GRPR). The GRPR cells are thought to be “tertiary pruritoceptors,” conveying itch information to lamina I projection neurons of the anterolateral system (ALS). Surprisingly, we recently found that GRPR-expressing neurons belong to a morphological class known as vertical cells, which are believed to transmit nociceptive information to lamina I ALS cells. Little is currently known about synaptic circuits engaged by the GRPR cells. Here we combine viral-mediated expression of PSD95-tagRFP fusion protein with super-resolution microscopy to reveal sources of excitatory input to GRPR cells. We find that they receive a relatively sparse input from peptidergic and non-peptidergic nociceptors in SDH, and a limited input from A- and C-low threshold mechanoreceptors on their ventral dendrites. They receive synapses from several excitatory interneuron populations, including those defined by expression of substance P, neuropeptide FF, cholecystokinin, neurokinin B, and neurotensin. We investigated downstream targets of GRPR cells by chemogenetically exciting them and identifying Fos-positive (activated) cells. In addition to lamina I projection neurons, many ALS cells in lateral lamina V and the lateral spinal nucleus were Fos-positive, suggesting that GRPR-expressing cells target a broader population of projection neurons than was previously recognised. Our findings indicate that GRPR cells receive a diverse synaptic input from various types of primary afferent and excitatory interneuron, and that they can activate ALS cells in both superficial and deep regions of the dorsal horn.

## Introduction

The spinal dorsal horn receives somatosensory input from primary afferent axons, and these arborise in a lamina-specific pattern (Todd, 2010; Abraira and Ginty, 2013; Braz et al., 2014). Most unmyelinated (C) afferents and many finely myelinated (Aδ) afferents are nociceptors or thermoreceptors, with some nociceptors also responding to stimuli that evoke itch, and therefore functioning as pruritoceptors. In addition, hairy skin is innervated by C afferents that respond to low-threshold mechanical stimuli (C-LTMRs). The C and Aδ afferents terminate mainly in the superficial dorsal horn (SDH, laminae I–II). In contrast, myelinated low-threshold mechanoreceptors (A-LTMRs) arborise in the deeper dorsal horn laminae (IIi–V).

Information carried by primary afferents is conveyed through complex synaptic circuits involving local interneurons and descending axons, before being transmitted to the brain via projection neurons belonging to the anterolateral system (ALS). ALS neurons are present at relatively high density in lamina I, and are scattered through laminae III–VI of the dorsal horn, the lateral spinal nucleus (LSN), and the ventral horn (Wercberger and Basbaum, 2019; Wang et al., 2022). Although ALS projection neurons form the main ascending output of the system, they only account for ∼1% of dorsal horn neurons (Chung et al., 1984; Todd, 2010). The remainder are interneurons, with axons that arborise locally. The majority of dorsal horn interneurons are excitatory (glutamatergic), and these can be assigned to several different populations. Electrophysiological studies have identified three distinctive classes among excitatory interneurons in lamina II: vertical, radial, and transient central cells (Grudt and Perl, 2002). It was also shown that vertical cells were presynaptic to lamina I ALS neurons (Lu and Perl, 2005), and that following peripheral nerve injury, loss of inhibition could open a circuit involving vertical cells that allowed input from A-LTMRs to reach these projection neurons, thus contributing to tactile allodynia (Lu et al., 2013).

Recent neurochemical and transcriptomic studies have revealed several populations among SDH excitatory interneurons (Häring et al., 2018; Sathyamurthy et al., 2018; Peirs et al., 2020; Polgár et al., 2023). We have identified seven largely non-overlapping classes among these neurons. Five of these are characterised by the expression of neuropeptides, cholecystokinin (CCK), neurotensin, neurokinin B (NKB), substance P and neuropeptide FF (NPFF), and one is defined by the presence of green fluorescent protein (GFP) in a transgenic mouse line in which this is expressed under control of the gastrin-releasing peptide (GRP) promoter (GRP-GFP cells) (Gutierrez-Mecinas et al., 2016a,2019a,2019b; Bell et al., 2020). The other population consists of cells that express *Grpr*, which codes for the gastrin-releasing peptide receptor (GRPR) (Polgár et al., 2023; Quillet et al., 2023). There is a clear relationship between these neurochemical populations and the classes identified by Grudt and Perl (2002), since many of the substance P-containing neurons are radial cells, the GRP-GFP neurons correspond to transient central cells, and GRPR- and NPFF-expressing cells represent two distinct types of vertical cell (Dickie et al., 2019; Polgár et al., 2023; Quillet et al., 2023). The other populations (those expressing CCK, NKB and neurotensin) are mainly located in the ventral part of lamina II, and include a class of neurons that were previously defined by their expression of the γ isoform of protein kinase C (PKCγ).

Gastrin-releasing peptide and the GRPR have attracted considerable attention, as they are both strongly implicated in itch. Mice lacking GRPR show reduced pruritogen-evoked itch, but normal pain behaviour, while intrathecal injection of a GRP agonist caused scratching that was blocked by administration of the antagonist (Sun and Chen, 2007). Consistent with this, ablation of GRPR cells reduced itch, but not pain behaviour (Sun et al., 2009). GRP released from local excitatory interneurons is thought to act on the GRPR neurons, which have been defined as “tertiary pruritoceptors” (Mishra and Hoon, 2013; Pagani et al., 2019). It is therefore surprising that the GRPR neurons correspond to vertical cells, as these are thought to form part of a pathway that transmits pain-related information (Lu et al., 2013). Interestingly, we recently showed that GRPR cells responded not only to pruritic stimuli, but also to mechanical and thermal noxious stimuli applied to the hindlimb, and that chemogenetic activation of GRPR cells in the lumbar spinal cord resulted in behaviours that appeared to reflect both itch and pain (Polgár et al., 2023). At present, little is known about the neuronal circuits involving the GRPR cells, although we have shown that they receive a powerful inhibitory input from NPY-expressing interneurons (Boyle et al., 2023). Here, we have investigated their excitatory synaptic inputs. We have also used chemogenetic activation of the GRPR cells to identify the ALS neurons that are included among their downstream targets.

## Materials and methods

### Animals and surgery

Experiments were approved by the Ethical Review Process Applications Panel of the University of Glasgow, and were performed in accordance with the UK Animals (Scientific Procedures) Act 1986. The study was carried out in compliance with the ARRIVE guidelines. All mice undergoing surgery (see below) received perioperative analgesia (0.1 mg/kg buprenorphine and 10 mg/kg carprofen). We used two mouse lines in which GRPR cells express recombinases: GRPR^*CreERT*2^ (Mu et al., 2017; Polgár et al., 2023) and GRPR*^Flp^* (Liu et al., 2019; Boyle et al., 2023). Since the Grpr gene is located on the X chromosome, all experiments with these lines used males that were hemizygous, or females that were homozygous, for the mutated allele.

Synaptic input to GRPR-expressing neurons was examined in the GRPR^*CreERT*2^ line. To visualise the GRPR-expressing cells and their excitatory synaptic input, we used two AAVs that encoded fluorescent proteins in a Cre-dependent manner (Table 1). One of these (AAV.flex.GFP) generated GFP and the other (AAV.flex.PSD95-tagRFP) caused expression of a fusion protein consisting of the postsynaptic density protein PSD95 and the red fluorescent protein tagRFP, thus allowing direct visualisation of excitatory synapses (Barik et al., 2018). These AAVs were co-injected into the right side of the spinal cord of 6 GRPR^*CreERT*2^ mice (either sex, 17–24 g), as described previously (Polgár et al., 2023). Briefly, the mice were anaesthetised with isoflurane (1–2%) and placed in a stereotaxic frame with vertebral clamps attached to the T12 and L1 vertebrae. Injections were made through a small slit in the dura into the L3 and L5 spinal segments via the T12/T13 and T13/L1 intervertebral spaces, respectively. A glass micropipette attached to a 10 μl Hamilton syringe was placed 400 μm lateral to the midline and advanced to a depth of 300 μm below the spinal cord surface. A total volume of 300 nl of virus was infused at each injection site at a rate of 30–40 nl per minute, using a syringe pump (Harvard Apparatus, MA, USA). The pipette was left in place for 5 min to minimise leakage, and then the wound was closed. The mice were given 1 or 2 doses of tamoxifen (3 mg in 0.15 ml corn oil, i.p.) 4–5 days after surgery, and survived for a further 2 weeks, before being deeply anaesthetised with pentobarbitone (20 mg i.p.) and perfused through the left cardiac ventricle with fixative that contained 4% freshly depolymerised formaldehyde.

**TABLE 1:** AAV vectors.

	Serotype	Promoter	Construct	Source	Catalogue number	Details of injection	
						Number of GCs	Volume (nl)
AAV.flex.eGFP	AAV8	CAG	eGFP	VVF Zurich	v158-8	2.58 × 10^8^	300
AAV.flex.PSD95-tagRFP	AAV9	hSyn1	PSD95-tagRFP	M. Hoon		4.95 × 10^9^	300
AAV.flex.hM3D(Gq)	AAV2	hSyn1	hM3Dq-mCherry	VVF Zurich	V89-2	3.38 × 10^8^	300
AAV.frt.hM3D(Gq)	AAV2	hSyn1	hM3Dq-mCherry	VVF Zurich	V189-2	6.60 × 10^8^	300

The values in columns 7 and 8 refer to individual injections; in most cases mice received more than one injection, into different segments and/or into different sides of the same segment. GC, gene copies.

To identify downstream targets of GRPR cells, we administered clozapine-N-oxide (CNO) under terminal general anaesthesia to mice in which the excitatory chemogenetic receptor hM3Dq (Armbruster et al., 2007) was expressed by these cells, and tested for Fos expression. Initially, this was performed on 5 GRPR^*CreERT*2^ mice (either sex, 17–22 g) that received intraspinal injections of AAV.flex.hM3Dq-mCherry (Table 1) into the L3, L4, and L5 spinal segments on the right side. The spinal injection was performed as described above, except that a small drill hole was made through the lamina of the T13 vertebra to allow access to the L4 spinal segment. The mice were given two doses of tamoxifen (3 mg) between the fifth and 7th post-operative days, and were allowed to survive for a further 17–23 days. They were then anaesthetised with either urethane or isoflurane and received an intraperitoneal injection of either 5 mg/kg CNO (three mice) or vehicle (saline, two mice). Anaesthesia was maintained for 2 h, and the animals were then perfused with fixative, as described above. In two additional mice, we tested whether ALS projection neurons were among those that expressed Fos after chemogenetic activation of GRPR cells. One of these was a GRPR^*CreERT*2^ mouse (female, 18 g) that had received an intraspinal injection of AAV.flex.hM3Dq-mCherry into the L3, L4, and L5 spinal segments on the right side, and two doses of tamoxifen (3 mg) 4–5 days later. The other mouse (male, 27 g) had been generated by crossing the GRPR*^Flp^* line with a Phox2a:Cre BAC transgenic mouse (Roome et al., 2020) and the Ai32 reporter line (Jackson Laboratory; Stock number 024109) in which Cre-mediated excision of a STOP cassette results in expression of the yellow fluorescent protein (YFP) fused to channelrhodopsin. In the resulting cross, YFP is present in the plasma membrane of Phox2a-derived ALS projection neurons (Alsulaiman et al., 2021), while GRPR cells express Flp recombinase. The GRPR*^Flp^*;Phox2a:Cre;Ai32 mouse received intraspinal injections (as described above) into the right side of the L3, L4, and L5 segments of AAV.frt.hM3Dq-mCherry (Table 1), which contains a Flp-dependent construct consisting of hM3Dq fused to mCherry. Between 2 and 5 weeks after the spinal injections, both mice received an injection of 300 nl of 0.1% cholera toxin B subunit (CTb) into the left lateral parabrachial area, as described previously (Alsulaiman et al., 2021). Three days after the CTb injections, they were anaesthetised with isoflurane and received an intraperitoneal injection of CNO (5 mg/kg). They were maintained under isoflurane anaesthesia for 2 h and then perfused with fixative, as described above.

### Tissue processing for immunohistochemistry

The L3 and L5 segments of spinal cords from the GRPR^*CreERT*2^ mice that had received spinal injections of AAV.flex.GFP and AAV.flex.PSD95-tagRFP were cut into 60 μm thick transverse sections with a vibrating blade microtome (Leica VT1000 or VT1200). These were incubated for 1–3 days in combinations of primary antibodies (listed in Table 2), which were diluted in phosphate buffered saline (PBS) that contained 0.3M NaCl, 0.3% Triton X-100 and 5% normal donkey serum. They were then incubated for 2–24 h in appropriate species-specific secondary antibodies, which were raised in donkey and conjugated to Alexa488, Alexa647, Rhodamine Red, Pacific Blue or biotin (all from Jackson ImmunoResearch, West Grove, PA, USA) or to Alexa Plus 405 (ThermoFisher Scientific). All secondary antibodies were diluted 1:500, except for Rhodamine Red, which was diluted 1:100. Biotinylated secondary antibodies were revealed with Pacific Blue conjugated to avidin (1:1,000; Life Technologies, Paisley, UK). To look for synaptic input from different types of primary afferent, sections were incubated in one of the following antibody combinations: (1) GFP (guinea pig) and prostatic acid phosphatase (PAP, a marker for non-peptidergic nociceptive afferents); (2) GFP (chicken), substance P and CGRP; (3) GFP (chicken), VGLUT3, and Homer; and (4) GFP (chicken) and VGLUT1. To look for synaptic input from interneurons, sections were reacted with mixtures of antibodies that included those against GFP (chicken or guinea pig) and VGLUT2 (rabbit or chicken) together with antibody against one of the following: pro-NPFF, preprotachykinin B (PPTB, the precursor for NKB), neurotensin, pro-CCK, or pro-GRP.

**TABLE 2:** Antibodies used in this study.

Antigen	Species	Dilution	Source; catalogue number or reference	RRID
GFP*	Chicken	1:1,000	Abcam; Ab13970	RRID:AB_300798
GFP*	Guinea pig	1:1,000	M. Watanabe	RRID:AB_2571575
Homer	Goat	1:1,000	M. Watanabe; Gutierrez-Mecinas et al. (2016b)	RRID:AB_2631104
VGLUT1	Rabbit	1:5,000	Synaptic systems; 135302	RRID:AB_887877
VGLUT2	Rabbit	1:5,000	Synaptic systems; 135402	RRID:AB_2187539
VGLUT2	Chicken	1:5,000	Synaptic systems; 135416	RRID:AB_2619824
VGLUT3	Guinea pig	1:100	M. Watanabe	RRID:AB_2571856
PAP	Chicken	1:1,000	Aves; PAP	RRID:AB_2313557
CGRP	Rabbit	1:5,000	Enzo; BML-CA1134	RRID:AB_2068527
Substance P	Rat	1:200	Oxford Biotechnology; OBT06435	
Pro-NPFF	Guinea pig	0.83 μg/ml	M. Watanabe; Gutierrez-Mecinas et al. (2019a)	RRID:AB_2783015
Pro-CCK	Rabbit	1:1,000	M. Watanabe	
Neurotensin	Rat	1:1,000	P. Ciofi; Porteous et al. (2011)	RRID:AB_2314928
PPTB	Guinea pig	1:1,000	P. Ciofi; Ciofi et al. (1994)	RRID:AB_2819032
Pro-GRP	Guinea pig	1:500	M. Watanabe; Gutierrez-Mecinas et al. (2023)	
mCherry	Rat	1:1,000	Invitrogen; M11217	RRID:AB_2536611
mCherry	Chicken	1:10,000	Abcam; Ab205402	RRID:AB_2722769
CTb	Goat	1:2,000^†^	List Biological; 703	RRID:AB_10013220
Fos	Goat	1:500	Santa Cruz; sc-52-G	RRID:AB_2629503
Phospho-c-Fos	Rabbit	1:500	Cell Signalling Technology; 5348	RRID:AB_10557109

*GFP antibodies also recognise YFP.

^†^CTb antibody used at 1:200,000 for immunoperoxidase staining of brain injection sites.

The L3–L5 segments of the five GRPR^*CreERT*2^ mice that received intraspinal injections of AAV.flex.hM3Dq-mCherry for chemogenetic activation (or control treatment) were cut into 60 μm thick parasagittal sections, which were reacted with antibodies against Fos and mCherry (chicken antibody). The L3–L5 segments of the two mice that received intraspinal injections of AAV.flex.hM3Dq-mCherry or AAV.frt.hM3Dq-mCherry, followed by brain injections of CTb were cut into 60 μm thick transverse sections, which were reacted with antibodies against CTb, GFP (chicken antibody, only for the GRPR*^Flp^*;Phox2a:Cre;Ai32 mouse), mCherry (rat antibody), and Phospho-c-Fos (Ser32). Brains of these mice were cut into 100 μm thick coronal sections, which were reacted to reveal CTb in the injection site by an immunoperoxidase method, as described previously (Alsulaiman et al., 2021).

### Analysis of synaptic inputs to GRPR cells

Tissue from the GRPR^*CreERT*2^ mice that had received intraspinal injections of AAV.flex.PSD95-tagRFP and AAV.flex.GFP was used to determine the proportion of excitatory synapses on GRPR cells at which the presynaptic profile contained markers of specific types of primary afferent or excitatory interneuron. For each of these markers, we examined two sections from each of three mice. These were scanned with a Zeiss LSM900 Airyscan confocal microscope equipped with 405, 488, 561, and 640 nm diode lasers in SR mode, and for each section confocal image stacks (0.5 μm z-separation) were obtained through a 63× oil-immersion lens (numerical aperture, NA 1.4) from three different regions across the mediolateral axis on the side ipsilateral to the spinal injections. In most cases, we restricted the analysis to laminae I and II, as the majority of dendrites belonging to GRPR cells are contained within this region (Polgár et al., 2023). For the interneuron markers the region analysed extended across the whole of laminae I and II, while for the primary afferent markers the analysis was limited to a region that included the main termination zone of the corresponding primary afferents (Table 3). Scans were analysed with Neurolucida software (MBF Bioscience, Williston, VT, USA). Initially, the channels corresponding to GFP and tagRFP were viewed, and a 3 μm × 3 μm grid was placed over the image stack. Tag-RFP puncta that were within GFP-positive profiles were analysed, as these correspond to postsynaptic densities at excitatory synapses on dendrites of GFP-labelled GRPR cells. A single optical section from within the z-stack was selected, and starting with a grid square at the dorsal surface of the grey matter, we marked the tag-RFP punctum within a GFP-positive profile that was nearest the right-hand side of the grid square. We then progressed through the squares in a dorsal-to-ventral and then left-to-right direction until we had acquired a minimum of 100 tag-RFP puncta from each region. If fewer than 100 puncta were obtained from this optical section, we moved to a different section at least 3 μm deeper in the tissue. We then revealed the remaining confocal channel(s) and noted the presence or absence of the other markers adjacent to each selected tag-RFP punctum.

**TABLE 3:** Quantitative analysis of synaptic inputs to GRPR cells.

Type	Immunohistochemical markers	Region analysed	Number of profiles	% with marker(s)
Primary afferent	SP+/CGRP+	I–II	1,271 (771–1,830)	2 (2–3)
	SP−/CGRP+	I–II	1,271 (771–1,830)	2 (1–4)
	PAP+	II	1,048 (682–1,607)	4 (3–5)
	VGLUT3+	IIi	411 (333–456)	4 (3–5)
	VGLUT1+	IIi–III	1,530 (931–2,541)	3 (2–3)
Excitatory interneuron	SP+/CGRP−	I–II	1,271 (771–1,830)	14 (13–15)
	Pro-NPFF+/VGLUT2+	I–II	691 (678–698)	8 (6–11)
	Pro-CCK+/VGLUT2+	I–II	709 (667–733)	16 (14–19)
	Neurotensin+/VGLUT2+	I–II	656 (642–684)	8 (6–9)
	PPTB+/VGLUT2+	I–II	622 (621–624)	17 (15–18)
	Pro-GRP+/VGLUT2+	I–II	654 (633–665)	15 (13–18)

Numbers in columns 4 and 5 are mean values from three mice, with ranges in brackets.

### Fos expression in projection neurons following chemogenetic activation of GRPR cells

Transverse sections from the two CTb-injected mice in which GRPR cells were chemogenetically activated were scanned with a Zeiss LSM710 confocal microscope equipped with Argon multi-line, 405 nm diode, 561 nm solid state and 633 nm HeNe lasers, through a 40× oil-immersion lens (NA 1.3). Confocal image stacks (2 μm z-separation) were obtained through the full thickness of the section to include the entire dorsal horn, on the side ipsilateral to the spinal injections and these were analysed with Neurolucida software. In both mice, we used the presence of CTb to identify projection neurons. In the GRPR*^Flp^*;Phox2a:Cre;Ai32 mouse, we also included YFP-labelled cells, irrespective of whether these were labelled with CTb. This approach allowed us to identify additional projection neurons that were not labelled from the LPb injection site. We initially viewed the channels corresponding to CTb and YFP (in the GRPR*^Flp^*;Phox2a:Cre;Ai32 mouse), and marked the locations of all projection neurons that were retrogradely labelled with CTb and/or were Phox2a-positive (YFP-labelled). We then viewed the phospho-Fos channel and recorded the presence or absence of staining in the nuclei of each of the selected projection neurons. We also examined mCherry labelling to confirm the extent of the spinal injection site and to look for possible expression in projection neurons. We allocated the projection neurons to five regions based on the description given in Kókai et al. (2022): (1) laminae I–II, (2) the LSN, (3) lateral lamina V (this included the reticulated area together with the immediately adjacent region in the non-reticulated part), (4) medial laminae III–V (other cells in the deep dorsal horn), and (5) the area around the central canal. This allocation was done before phospho-Fos staining was examined.

### Characterisation of antibodies

The GFP and mCherry antibodies were raised against full-length recombinant proteins, and specificity is demonstrated by the absence of staining in tissue that lacks the corresponding protein. The Homer antibody was raised against amino acids 1–175 of the mouse Homer1 protein and detects bands of the appropriate size in immunoblots of mouse brain extracts (Gutierrez-Mecinas et al., 2016b). The VGLUT1 and VGLUT2 antibodies were raised against peptides corresponding to the C terminal part of the rat protein. The VGLUT1 antibody shows no staining in mice lacking the VGLUT1 protein (manufacturer’s specification), while the VGLUT2 antibodies show the same staining pattern as other well-characterised antibodies against VGLUT2. The VGLUT3 antibody was raised against amino acids 522–588 of the mouse protein, and detects a single band at 60–62 kDa. The PAP antibody was raised against recombinant mouse PAP, while the CGRP antibody was raised against a synthetic peptide corresponding to a portion of the rat α-calcitonin gene-related peptide, and in both cases the staining with these antibodies closely matches that reported in the literature. The monoclonal substance P antibody detects the C-terminal 5–8 amino acids of substance P and does not appear to recognise NKB (McLeod et al., 2000). The pro-NPFF, and pro-GRP antibodies were raised against amino acids 22–114 of mouse pro-NPFF and 53–84 of mouse pro-GRP (Gutierrez-Mecinas et al., 2019a,^2023^). The pro-CCK antibody was raised against amino acids 67–87 of mouse pro-CCK and gives the expected staining pattern in the hippocampus, with dense basket-like arrangement around pyramidal cell somata, and dense labelling in the innermost layer of dentate gyrus molecular layer (MW unpublished observations). The PPTB antibody was raised against amino acids 50–79 from rat PPTB conjugated to human serum albumin, and immunostaining is blocked by pre-incubation with the immunising peptide, but not with substance P or neurokinin A (Ciofi et al., 1994). Staining with the rat polyclonal neurotensin antibody is blocked by pre-incubation with the peptide (Porteous et al., 2011). The CTb antibody was raised against the purified protein, and specificity is demonstrated by the lack of staining in regions that did not contain injected or transported tracer. The goat antibody against Fos was raised against a peptide corresponding to the N-terminus of human Fos, while the phospho-Fos antibody is a rabbit monoclonal, and recognises the c-Fos protein phosphorylated at Ser32. Specificity of the phospho-Fos antibody is demonstrated by immunoblots of extracts from HeLa cells that were stimulated in the presence or absence of λ-phosphatase (manufacturer’s specification). In addition, specificity of both Fos antibodies is shown by the restriction of labelling to specific neuronal populations following chemogenetic activation of the GRPR cells.

### Fluorescence *in situ* hybridisation

Fluorescence *in situ* hybridisation (FISH) was performed with RNAscope probes and RNAscope fluorescent multiplex reagent kit 320850 (ACD BioTechne; Newark, CA 94560). Fresh frozen lumbar spinal cords from three wild-type mice (both sexes, 20–25 g) were embedded in OCT medium and cut into transverse 14 μm thick sections with a cryostat (Leica CM1950; Leica, Milton Keynes, UK). The sections were mounted onto SuperFrost Plus slides (48311-703; VWR; Lutterworth, UK) such that each section on a slide was at least 4 apart in the z-series. Reactions were performed according to the manufacturer’s recommended protocol. Sections were reacted with probes for *Grpr*, *Slc17a6*, and *Homer1*, and details of the probes are listed in Table 4. Sections were mounted with Prolong-Glass anti-fade medium with NucBlue (Hoechst 33342; ThermoFisher Scientific, Paisley, UK). The full thickness of the section was scanned with the Zeiss LSM 710 confocal microscope through a 40× oil-immersion lens to cover the whole of laminae I–II.

**TABLE 4:** RNAscope probes used in this study.

Probe	Protein/peptide	Channel numbers	Catalogue numbers	Z-pair number	Target region
*Grpr*	GRPR	3	317871	20	463–1,596
*Slc17a6*	VGLUT2	2	319171	20	1,986–2,998
*Homer1*	Homer1	1	557951	16	1,301–2,197

We performed cell counts to compare the level of expression of *Homer1* in *Grpr*-positive and *Grpr*-negative excitatory neurons, which were identified by the presence of *Slc17a6*. Quantitative analysis of transcript numbers was conducted using the cell detection and subcellular objects features of QuPath software (Bankhead et al., 2017). Maximum projections obtained from confocal image stacks were used. Recognition and segmentation of individual nuclei was performed based on NucBlue staining and an additional 2 μm perimeter was added to each nucleus to allow detection of perinuclear transcripts. Any areas with poor nuclear segmentation were manually excluded from the analysis. Detection thresholds were adjusted manually until the mark-up accurately reflected the transcript distribution; then a transcript count for each individual cell was obtained. Cells were defined as positive for expression of *Grpr* or *Slc17a6* if they contained four or more transcripts, while the number of *Homer1* transcripts was recorded for each *Slc17a6*-positive cell.

### Statistics

The Mann–Whitney *U* test was used to compare *Homer1* transcript numbers in *Grpr*-positive and *Grpr*-negative cells. To determine whether there was a significant difference between the synaptic input to GRPR cells arising from primary afferents versus excitatory interneurons, we used a mixed effects linear model in Jamovi (Version 2.25, The Jamovi Project). Each marker examined was allocated to either primary afferent or interneuron categories and the percentage input for each marker calculated per animal. The percentage of synaptic input was used as the dependent variable, with interneuron vs. primary afferent used as a factor and animal entered as the cluster variable.

## Results

### Homer as a marker for excitatory synapses on GRPR cells

**FIGURE 1:**
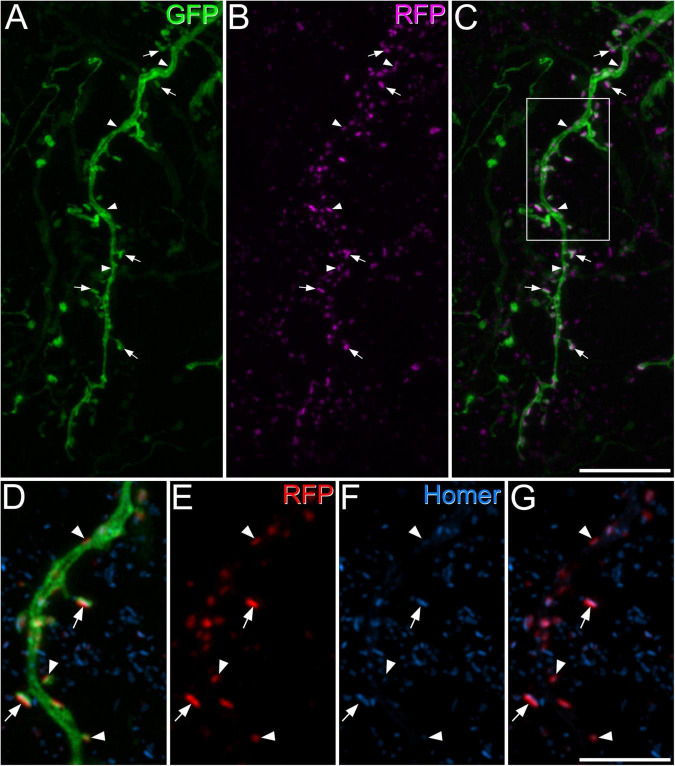
Green fluorescent protein, tagRFP, and Homer expression in a GRPR^CreERT2^ mouse that had received intraspinal injection of AAV.flex.GFP and AAV.flex.PSD95-tagRFP. **(A)** A dendrite labelled with GFP (green) extends through part of lamina II. **(B)** Numerous tagRFP-labelled puncta RFP, (magenta) are also present in this region. **(C)** A merged image shows that most of these tagRFP puncta are associated with the GFP-labelled dendritic shaft and its spines. Some of the tagRFP puncta in dendritic spines are marked with arrows, and some of those in dendritic shafts with arrowheads. The box in panel **(C)** indicates the region shown in more detail in panels **(D–G)**. **(D–G)** A higher magnification view through part of the dendrite stained to reveal GFP (green), tagRFP (red) and Homer (blue). Some of the tagRFP puncta associated with the labelled dendrite have relatively strong staining for Homer (two marked with arrows), while in others the Homer staining is barely detectable (three marked with arrowheads). This field also contains numerous Homer puncta that are not associated with tagRFP-labelling, and these are presumably located in other neurons in the vicinity. Images are projections of 43 **(A–C)** and 6 **(D–G)** optical sections at 0.3 μm z-spacing. Scale bars: 10 μm **(A–C)** and 5 μm **(D–G)**.

Homer has been used as a convenient marker for excitatory synapses, as it can be revealed in studies that use multiple immunofluorescence labelling to examine synaptic connectivity (Gutierrez-Mecinas et al., 2016b; Abraira et al., 2017; Larsson and Broman, 2019; Polgár et al., 2020). However, in preliminary studies, we had observed that dendritic spines belonging to the GRPR cells often lacked detectable Homer immunoreactivity, suggesting that Homer antibodies may fail to detect some excitatory synapses on these cells. We therefore tested an alternative approach, in which AAV coding for Cre-dependent PSD95-tagRFP fusion protein is used to identify postsynaptic densities at excitatory synapses (Barik et al., 2021). When this was injected intraspinally, together with AAV.flex.GFP, into GRPR^*CreERT*2^ mice we saw a similar pattern of GFP labelling to that described by Polgár et al. (2023), with cell bodies that were present at the highest density in laminae I and IIo, and dendrites that extended ventrally throughout the SDH. This pattern also closely resembles the labelling seen when the GRPR^*CreERT*2^ line is crossed with a Cre-dependent reporter mouse. TagRFP-labelling was mainly seen in the SDH, consistent with expression by GRPR-positive neurons (Supplementary Figure 1). At high magnification, tagRFP was in the form of small puncta, many of which were associated with GFP-labelled dendrites, and these could be located either in dendritic spines or shafts (Figures 1A–C). In sections that had also been stained for Homer, we found that many of the tagRFP profiles were also Homer immunoreactive. However, in many of the tagRFP puncta Homer immunoreactivity was very weak, and in some cases it was barely detectable (Figures 1D–G). Most of the dendritic spines attached to GFP-labelled dendritic shafts contained at least one tagRFP punctum. We noted that some tagRFP profiles were not associated with GFP-labelled dendrites, and these are likely to be in GRPR-expressing cells that were not transfected with the AAV.flex.GFP virus. In some cases, we observed GFP-labelled dendrites that contained Homer puncta, but lacked tagRFP, and presumably this reflects transfection with only the AAV.flex.GFP vector.

### Fluorescence *in situ* hybridisation

**FIGURE 2:**
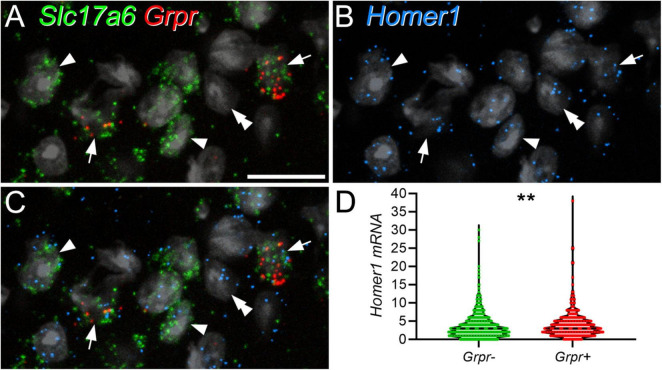
*Homer1* expression in GRPR-positive and GRPR-negative excitatory neurons. Panels **(A–C)** show confocal images from lamina II in a section that had undergone a fluorescent *in situ* hybridisation reaction to reveal *Slc17a6* (green), *Grpr* (red), and *Homer1* (blue) mRNAs. The section has been stained with the nuclear stain NucBlue, which is shown in grey. Excitatory neurons are revealed by expression of *Slc17a6*, which codes for VGLUT2, and 2 of these (arrows) are *Grpr*-positive. Two of the *Grpr*-negative excitatory (*Slc17a6*-positive) cells are marked with arrowheads, and a *Slc17a6*-negative cell with a double arrowhead. Transcripts for *Homer1* are present over all of these cells. **(D)** Violin plot of *Homer1* transcript number in *Grpr*-negative and *Grpr*-positive cells. Transcript number was significantly higher in *Grpr*-positive cells (**p = 0.0011; Mann–Whitney *U* test). Dashed lines represent the median values. Images in panels **(A–C)** are maximum intensity projections through the full thickness of the section, obtained at 0.5 μm z-spacing. Scale bar: 20 μm.

As noted above, we found that GRPR cells often had very low levels of Homer immunoreactivity at putative excitatory synapses, and interestingly, Sathyamurthy et al. (2018) found the lowest level of average gene expression for *Homer1* in their DE12 cluster, which corresponds to GRPR-expressing cells. We therefore used FISH to determine whether the GRPR-positive neurons contained fewer *Homer1* transcripts than GRPR-negative excitatory cells (Figure 2). *Homer1* transcripts were widely distributed on cells in the dorsal horn, including those with *Grpr*. The mean *Homer1* transcript number for *Grpr*-positive cells was 3.97 ± 3.7 (SD), while that for *Grpr*-negative excitatory neurons was 3.4 ± 3.2, and surprisingly, we found that the transcript number was significantly higher for the *Grpr*-positive cells (*p* = 0.0011; Mann–Whitney *U* test). Lack of *Homer1* mRNA therefore does not explain the relatively low level of Homer-immunoreactivity at many of the excitatory synapses on these cells.

### Quantitative analysis of synaptic inputs to GRPR cells

In sections taken from GRPR^*CreERT*2^ mice that had received intraspinal injections of AAV.flex.GFP and AAV.flex.PSD95-tagRFP we found a very high density of GFP-labelled dendrites, in laminae I-II consistent with our previous findings (Polgár et al., 2023). This made it impossible to reconstruct dendritic trees of individual cells, and we therefore analysed synaptic inputs to GFP-labelled dendritic or somatic profiles without attempting to assign them to particular neurons. The great majority of GRPR cells in the dorsal horn are located in lamina I and the most superficial part of lamina II, and we have found, using the Brainbow technique, that these were all vertical cells (Polgár et al., 2023). However there are also a few GRPR cells in deeper parts of the dorsal horn, and these can differ in morphology. Nonetheless, the vast majority of the dendrites analysed are likely to have originated from GRPR-expressing vertical cells in laminae I–IIo, since these are far more numerous than GRPR cells in deeper laminae.

**FIGURE 3:**
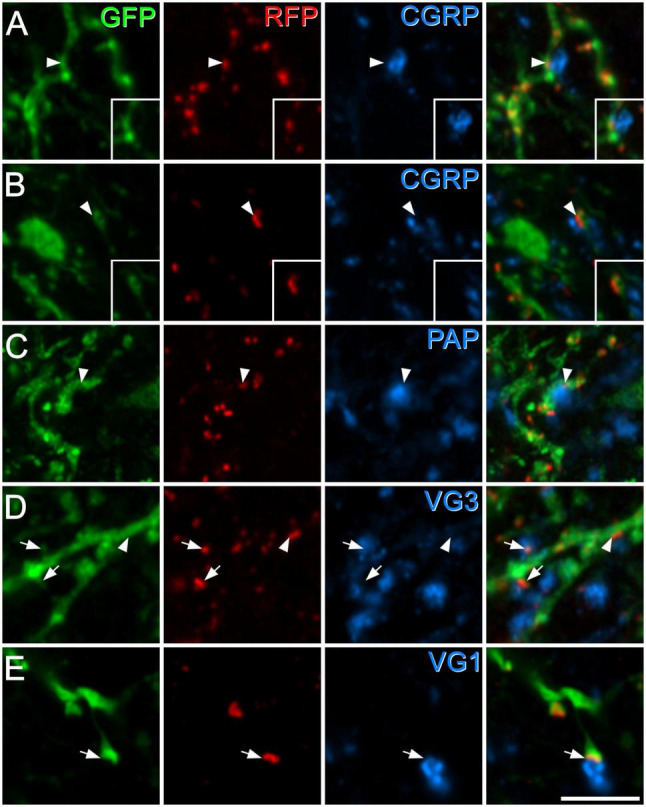
Synapses from primary afferent axons onto GRPR cells. Panels **(A,B)** show examples of synapses formed by peptidergic afferents that contain both CGRP and substance P **(A)**, or CGRP but not substance P **(B)**. In each case, GFP-labelling (green) in GRPR neurons is shown in the left pane, tagRFP (RFP, red) in the second pane and CGRP (blue) in the third pane, while the fourth pane is a merged image. In the insets, staining for substance P (blue) is shown instead of staining for CGRP. In each case, the arrowhead marks a tagRFP punctum (site of excitatory postsynaptic density) in a GFP-labelled dendrite that is in contact with a CGRP-immunoreactive bouton. In panel **(A)**, this is a large bouton that lies to the right of the tagRFP punctum and is also immunoreactive for substance P. In panel **(B)**, a small CGRP bouton lies to the left of the tagRFP punctum and is not substance P-immunoreactive. Panels **(C–E)** show examples of synapses from boutons that are immunoreactive for PAP **(C)**, VGLUT3 (VG3, **D**), or VGLUT1 (VG1, **E**). In each case the layout is the same as that in the main parts of panels **(A,B)**. Arrowheads indicate tagRFP puncta in GFP-labelled dendritic shafts that are in contact with an immunoreactive bouton, while arrows mark tagRFP puncta in GFP-labelled dendritic spines that are contacted by immunoreactive boutons. Images are maximum intensity projections from 2 **(B,D,E)** or 3 **(A,C)** optical sections at 0.5 μm z-separation. Scale bar: 5 μm.

The dendrites of GRPR cells could potentially receive monosynaptic input from several classes of primary afferent. To identify peptidergic nociceptors, we used antibodies against substance P and CGRP, while non-peptidergic nociceptors were identified with antibody against PAP (Usoskin et al., 2015; Bell et al., 2020; Qi et al., 2023). A and C low-threshold mechanoreceptors were identified with antibodies against VGLUT1 and VGLUT3, respectively (Todd et al., 2003; Seal et al., 2009). We found that primary afferent boutons identified with these antibodies accounted for a relatively low proportion of the excitatory synapses on the GRPR cells (Table 3 and Figure 3). We identified two types of peptidergic afferent, those with both CGRP and substance P, and those with only CGRP. In both cases, these were associated with 2% of the tagRFP puncta on processes of GRPR cells in laminae I–II. Non-peptidergic (PAP+) inputs were only quantified within the band of PAP-immunoreactive profiles, which occupies the middle part lamina II, and within this region, 4% of tagRFP labelled synapses in GFP profiles were contacted by PAP+ boutons. C-LTMRs, identified by expression of VGLUT3, form a plexus within lamina IIi, and within this region they accounted for 4% of the excitatory synaptic input to GRPR cells. A-LTMRs arborise in a broad area extending from lamina IIi through to lamina V, and these all express VGLUT1 (Todd et al., 2003). Although VGLUT1 is also present in corticospinal terminals, these are much smaller than the A-LTMR boutons, and we therefore quantified contacts from large VGLUT1 profiles within a region that extended from lamina IIi through to lamina III. Within this region VGLUT1-immunoreactive boutons accounted for 3% of the excitatory synapses on GRPR cells. Importantly, since we only analysed input from non-peptidergic nociceptors and LTMRs within the relatively narrow region in which these afferents overlap with GRPR dendrites, the actual proportion of excitatory synaptic input that they provide to GRPR cells will be considerably lower than the values that we report here. Overall, these results demonstrate that although primary afferents provide monosynaptic input to the GRPR cells, this accounts for only a small minority of their excitatory synapses.

**FIGURE 4:**
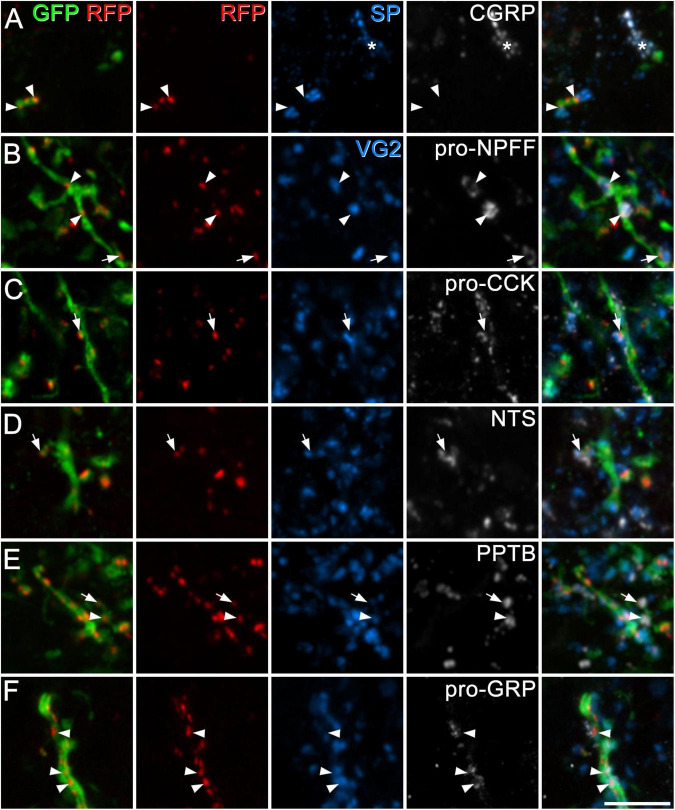
Synapses from axons of dorsal horn excitatory neurons onto GRPR cells. In each case, the first pane contains a merged image showing GFP (green) and tagRFP (RFP, red) to show the locations of excitatory postsynaptic densities in the dendrites of GRPR neurons, while the second pane shows just the tagRFP puncta, to allow clear visualisation of these structures. The fifth pane is a merged image showing all four confocal channels. In panel **(A)**, the third and fourth panes show substance P (SP, blue) and CGRP (grey), and two substance P-immunoreactive boutons that lack CGRP contact tagRFP puncta (arrowheads) on a GFP-labelled dendrite. The asterisk indicates a group of profiles that contain both substance P and CGRP (derived from peptidergic nociceptors). In panels **(B–F)** the third pane shows immunostaining for VGLUT2 (VG2, blue), and the fourth pane immunostaining for peptides or propeptides that are expressed by dorsal horn excitatory neurons: pro-NPFF, pro-CCK, neurotensin (NTS), preprotachykinin B (PPTB), and pro-GRP, respectively. Arrowheads and arrows indicate tagRFP puncta on GFP-labelled dendritic shafts and dendritic spines (respectively) that are in contact with boutons that are immunoreactive for VGLUT2 and the corresponding peptide/propeptide. Images are maximum intensity projections from 2 **(C,D)**, 3 **(F)**, 4 **(B,E)**, or 6 **(A)** optical sections at 0.5 μm z-separation. Scale bar: 5 μm.

We used antibodies against several neuropeptides (or neuropeptide precursors) that are associated with specific subpopulations of excitatory interneurons in laminae I–II (Gutierrez-Mecinas et al., 2016a; Häring et al., 2018; Quillet et al., 2023). Specifically, we combined immunostaining for VGLUT2 (which is expressed in all dorsal horn excitatory neurons) with detection of pro-CCK, neurotensin, PPTB or pro-NPFF, to reveal axons originating, respectively, from cells in the Glut1–3, Glut4, Glut5–7, and Glut9 populations of Häring et al. (2018). To identify axons of substance P-expressing neurons (Glut10–11), we used tissue reacted with antibodies against substance P and CGRP, and quantified contacts from substance P-positive/CGRP-negative boutons. This approach was used in order to exclude substance P-containing nociceptive primary afferents, which co-express CGRP (Usoskin et al., 2015; Qi et al., 2023). Finally, we used an antibody against the precursor for GRP (pro-GRP) (Gutierrez-Mecinas et al., 2023). Although GRP is widely expressed among the excitatory interneuron populations identified by Häring et al. (Glut5–12), and therefore does not define a specific population, it is the canonical ligand for GRPR. We found that pro-NPFF- and neurotensin-immunoreactive axons each accounted for 8% of the excitatory synapses on GRPR cells in laminae I–II, while between 14 and 17% of these synapses originated from axons belonging to each of the other populations (defined by expression of substance P, pro-CCK, PPTB, and pro-GRP) (Table 3 and Figure 4). Overall, it appeared that more synaptic input to GRPR cells was derived from excitatory interneurons that primary afferents, and this effect was highly significant (*p* < 0.001).

**FIGURE 5:**
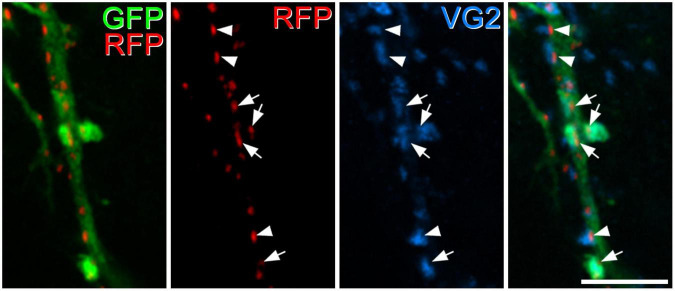
Excitatory synapses from axons of GRPR cells onto dendrites of GRPR cells. In this image, the first pane contains a merged image of GFP (green) and tagRFP (RFP, red) to show the locations of excitatory postsynaptic densities in the dendrite of a GRPR neuron, while the second pane shows just the tagRFP puncta, to allow clear visualisation of these structures. The third pane shows immunostaining for VGLUT2 (VG2, blue), and the fourth pane a merged image. A large GRP-labelled dendritic shaft passes vertically through the image, and this is associated with numerous tagRFP puncta. Four of these (marked by arrows) are contacted by profiles that are immunoreactive for VGLUT2 and GFP, and these represent boutons derived from axons of GRPR cells. Several other tagRFP puncta (3 marked by arrowheads) are associated with VGLUT2 boutons that lack GFP. The images are maximum intensity projections from 3 optical sections at 0.5 μm z-separation. Scale bar: 5 μm.

We have previously shown that GRPR cells receive excitatory synapses from other GRPR cells (Polgár et al., 2023). Consistent with this, we identified tagRFP-labelled puncta on dendrites of GFP-labelled cells that were in contact with profiles that were immunoreactive for both GFP and VGLUT2, and therefore correspond to axons of GRPR cells (Figure 5). We were not able to quantify the input from other GRPR cells, since not all of these cells would have been labelled by the viral injection.

### Downstream targets of GRPR cells

**FIGURE 6:**
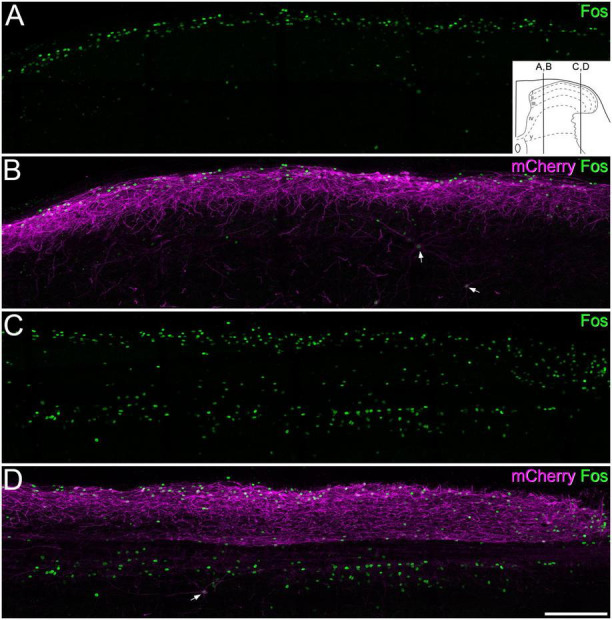
Fos expression following chemogenetic activation of GRPR cells. Panels **(A,B)** show immunostaining for Fos (green) and mCherry (magenta) in a sagittal section through the medial part of the dorsal horn of a GRPR^CreERT2^ mouse on the side ipsilateral to a spinal injection of AAV.flex.hM3Dq-mCherry. The mouse received an intraperitoneal injection of clozapine-N-oxide (5 mg/kg) while under general anaesthesia, which was maintained for 2 h prior to perfusion fixation. There is a dense band of mCherry labelling in the superficial laminae, corresponding to the cell bodies and dendrites of GRPR cells. Fos-positive nuclei are largely restricted to this region, with only a few in deeper laminae. mCherry-positive cells are occasionally seen in deeper laminae. Two of these are indicated with arrows, and these are Fos-positive. **(C,D)** A section through the lateral part of the dorsal horn from the same animal. The mCherry labelling is again largely restricted to the superficial dorsal horn, and this region contains Fos-labelled nuclei. However, there are also many Fos-positive nuclei in the deeper part of the dorsal horn, in an area corresponding to the lateral, reticulated part of lamina V. The great majority of these lack mCherry-labelling, although one deep mCherry-positive cell, which is also Fos-positive, is present (arrow). The inset in panel **(A)** shows the approximate mediolateral positions of the sagittal sections shown in A, B and C, D represented on a transverse section of the lumbar dorsal horn. Images are maximum intensity projections through the full thickness of a 60 μm section. Scale bar: 200 μm.

In initial experiments, five GRPR^*CreERT*2^ mice that had received intraspinal injections of AAV.flex.hM3Dq-mCherry were given CNO (*n* = 3) or vehicle (*n* = 2) and were maintained under anaesthesia for 2 h. We observed a dense band of mCherry-labelled cells that were largely restricted to the dorsal horn with only a few labelled cells in deeper laminae, as described previously (see Figure 12 of Polgár et al., 2023). Surprisingly, we found that in the mice treated with CNO the pattern of Fos labelling differed between medial and lateral parts of the dorsal horn (Figure 6). In sections through the medial part, Fos-immunoreactive cells were numerous in the superficial laminae, and many of these cells were also mCherry-positive. In these sections, relatively few Fos-positive cells were seen in deeper laminae. However, in sections through the lateral part of the dorsal horn an additional band of Fos-positive cells (the vast majority of which lacked mCherry) was seen between 200 and 350 μm below the dorsal surface of the grey matter, corresponding to the lateral part of lamina V. In the mice that received vehicle, there were many fewer Fos-positive cells (Supplementary Figure 2).

**FIGURE 7:**
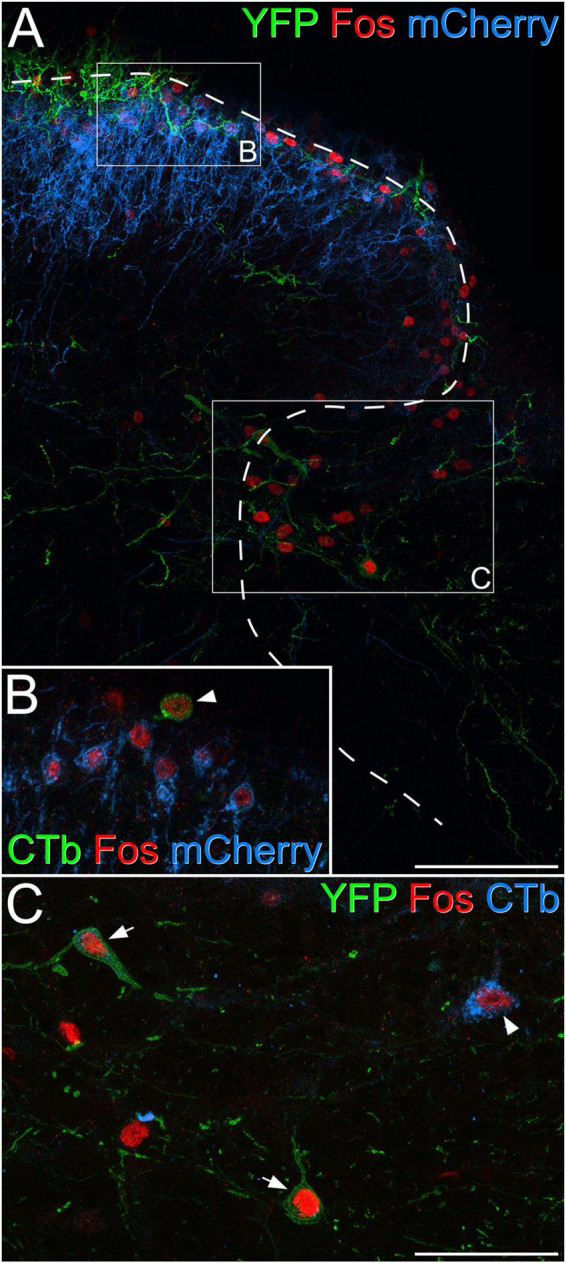
Fos expression in projection neurons following chemogenetic activation of GRPR cells. **(A)** Part of the dorsal horn of the L4 segment from a GRPR^Flp^;Phox2a:Cre;Ai32 mouse that had had an intraspinal injection of AAV.frt.hM3Dq-mCherry and brain injection of CTb. The mouse received an intraperitoneal injection of 5 mg/kg clozapine-N-oxide under general anaesthesia, which was maintained for 2 h prior to perfusion fixation. mCherry labelling (blue) is associated with GRPR cells, which are largely restricted to the superficial laminae, while processes belonging to YFP-labelled (green) Phox2a-derived projection neurons are present in lamina I and scattered through the deeper laminae. Many Fos-positive (red) nuclei can be seen in laminae I-IIo as well as in the lateral part of lamina V. Boxes indicate the regions shown at higher magnification in panels **(B,C)**, while the dashed line shows the border of grey matter. **(B)** Several nuclei that are immunoreactive for Fos (red) are visible. Most of these belong to GRPR cells that show mCherry-labelling (blue) in the cell membrane, while one (arrowhead) belongs to a lamina I projection neuron that is retrogradely with CTb (green) and lacks mCherry. **(C)** The lateral reticulated part of lamina V also contains many Fos-positive (red) nuclei, and three of these belong to projection neurons identified by labelling with CTb (blue, arrowhead) or expression of YFP (green), indicating that they are Phox2a-lineage cells (arrows). Scale bars: panel **(A)**, 100 μm, panels **(B,C)**, 50 μm. Images in panels **(A–C)** are maximum projections of 31, 2, and 3 optical sections, respectively, all with a z-separation of 2 μm.

To determine whether the Fos-positive cells included projection neurons belonging to the ALS we examined sections from the two mice that had received injections of CTb into the LPb (Supplementary Figure 3) and underwent chemogenetic activation of GRPR cells (Figure 7 and Table 5). Surprisingly, we found that relatively few lamina I projection neurons (9%) were Fos-positive. In contrast, the proportions of projection neurons with Fos were considerably higher in the LSN (46%) and the lateral part of lamina V (62%). In addition, some projection neurons in the medial part of the deep dorsal horn or around the central canal were Fos-positive (15 and 21%, respectively). Fos cells were much more rarely seen on the contralateral side (Supplementary Figure 4). The vast majority of projection neurons, including all of those in the SDH, the LSN, the medial part of the deep dorsal horn and the area around the central canal lacked mCherry, while a very small proportion of those in lateral lamina V (four cells in each animal, corresponding to between 5 and 7% of ALS cells in this region) were mCherry-labelled, and these were also Fos-positive.

**TABLE 5:** Phospho-Fos in projection neurons following chemogenetic activation of GRPR cells.

Region	Number of projection neurons	Number of projection neurons that are Phospho-Fos+	% of projection neurons that are Phospho-Fos+
Laminae I–II	91.5 (52, 131)	10 (2, 18)	8.8% (3.8%, 13.7%)
LSN	32.5 (28, 37)	15.5 (9, 22)	45.8% (32.1%, 59.5%)
Lateral lamina V	67 (75, 59)	41.5 (44, 39)	62.4% (58.7%, 66.1%)
Medial laminae III–V	20.5 (14, 27)	3 (2, 4)	14.6% (14.3%, 14.8%)
Central canal	48.5 (60, 37)	11 (16, 6)	21.4% (26.7%, 16.2%)

This table shows results from the two mice that received injections of CTb into the lateral parabrachial area and underwent chemogenetic activation of GRPR cells. Numbers in columns 2–4 are means, with individual values in brackets (GRPR^CreERT2^ mouse shown first).

To look for possible direct synaptic input from GRPR cells to projection neurons in the LSN and the lateral part of lamina V, we examined sections through these regions from GRPR^CreERT2^;Ai9 mice that had received injections of AAV.GFP into the lateral parabrachial area, as part of a separate study. The sections had been immunoreacted to reveal tdTomato, GFP and Homer. We found several examples of GFP-labelled dendrites that were in contact with tdTomato-positive boutons with an intervening Homer punctum, indicating the presence of an excitatory synapse (Supplementary Figure 5), although it was generally not possible to follow these dendrites to their cell bodies, due to the size of the dendritic trees of projection neurons.

Finally, to test whether GRPR cells located in the SDH had axons that could be followed into either the LSN or the deeper laminae of the dorsal horn, we examined archived material from slices taken from GRPR^CreERT2^ mice in which electrophysiological recordings had been made from Cre-positive cells, as described in Polgár et al. (2023). For cells that had been patched in transverse slices, we identified a few cases in which the axon of the patched cell could be followed into the LSN and/or the lateral part of lamina V (Supplementary Figure 6).

## Discussion

Our main findings are that: (1) although GRPR cells apparently have low levels of expression of Homer, their excitatory synapses can be revealed by injecting an AAV coding for PSD95-tagRFP; (2) GRPR cells are predominantly innervated by excitatory interneurons, and receive a relatively sparse synaptic input from primary afferents; and (3) chemogenetic excitation of these cells results in activation of other dorsal horn neurons, including ALS projection neurons, particularly those located in lateral lamina V and the LSN.

### Homer expression in GRPR cells

In preliminary studies, we had found that although Homer puncta could be detected on dendritic shafts and spines of GRPR cells, these were typically very weakly labelled, in comparison to Homer puncta nearby that were not associated with GRPR cells, and in some cases dendritic spines of GRPR cells lacked a clear Homer punctum. This is consistent with our previous finding that Homer antibodies do not reveal all excitatory synapses (Gutierrez-Mecinas et al., 2016b). The relatively weak immunostaining for Homer at synapses on GRPR cells is unlikely to be due to a lack of *Homer* mRNA in these cells, since the transcript numbers were significantly higher than those seen on other excitatory neurons. However, it is possible that failure to translate the mRNA, or rapid degradation of either mRNA or protein (Vogel and Marcotte, 2012) would account for the low level of Homer protein seen at excitatory synapses on these cells. Although there are three separate families of Homer proteins derived from different genes (*Homer1*, *Homer2*, and *Homer3*), each of which gives rise to several splice variants, the first 120 amino acids (which are included in the antigen used to generate this antibody) are highly conserved, and the antibody is therefore likely to detect all forms of Homer proteins. The apparently low levels of Homer might result from masking of antigenicity at synapses on the GRPR cells, or may reflect some functional difference between excitatory synapses on the GRPR cells compared to those on other neurons. We have compared the amplitude of miniature EPSCs on GRPR cells with those on another population of vertical cells, those that contain pro-NPFF (Quillet et al., 2023), and found that the mean amplitude for GRPR cells was only slightly lower than that for the NPFF cells (30.1 vs. 34.3 pA). Homer puncta are clearly visible at synapses on the NPFF cells (Raphaëlle Quillet, unpublished observations), and this suggests that the low level of Homer protein detected at synapses on GRPR cells does not reflect a substantial difference in synaptic function between these two populations. Future studies of the distribution of glutamate receptor subunits would be needed to show whether receptor composition at glutamatergic synapses differs between GRPR cells and other types of SDH interneuron.

### Synaptic input to GRPR cells

The low level of Homer immunoreactivity at many synapses on GRPR cells made it difficult to analyse patterns of excitatory synaptic input onto these cells, and we therefore used AAV.flex.PSD95-tagRFP to label synapses (Barik et al., 2021). An additional advantage of this approach is that because tagRFP expression is restricted to Cre-positive cells, it allows unequivocal identification of synapses belonging to the cell being analysed. In contrast, we have found that when using Homer to identify synapses, it is not always possible to be sure that a particular Homer punctum belongs to the cell being examined (Kókai et al., 2022). Because of the dense plexus of GFP-labelled dendrites, we were not able to analyse entire dendritic trees of individual neurons, and so we could not directly determine the proportions of excitatory synapses derived from each source. However, a benefit of this strategy was that it allowed us to quantify input from multiple presynaptic sources on tissue from a relatively small number of mice.

For the primary afferent markers, we restricted our analysis to regions where these overlapped with dendrites of GRPR cells. These dendrites can extend for at least 100 μm in the dorsoventral axis (Polgár et al., 2023), whereas the plexuses of PAP- and VGLUT3-labelled boutons are each ∼30 μm thick. As noted above, this will mean that we considerably over-estimated the proportion of excitatory synapses originating from non-peptidergic nociceptors and C-LTMRs. A similar consideration applies to A-LTMRs, due to the limited overlap between the VGLUT1 plexus and GRPR dendrites. For the peptidergic primary afferents and excitatory interneuron populations, we analysed dendrites throughout laminae I–II, and the proportions that we found are therefore likely to be more representative. Although our analysis covered most types of primary afferent, we did not examine those expressing Trpm8 (Sharma et al., 2020; Qi et al., 2023). However, these are unlikely to account for a large proportion of the excitatory synaptic input to GRPR cells, since they are restricted to lamina I. In addition, we found that only two out of eight GRPR cells tested showed an increase in mEPSC frequency in response to application of the Trpm8 agonist WS-12 (Polgár et al., 2023).

The main finding in this part of the study was that although GRPR cells received synapses from several types of primary afferent neurons, these only accounted for a small proportion of the excitatory synaptic input to these cells. In contrast, the GRPR cells received a more substantial input from non-primary axonal boutons that expressed a variety of neuropeptides or their precursors, including substance P, PPTB, pro-CCK (14–17%), and to a lesser extent pro-NPFF and neurotensin (8% in each case). Importantly, these peptidergic markers are associated with largely non-overlapping populations of dorsal horn excitatory interneurons (Gutierrez-Mecinas et al., 2014, 2016a,2019a,2019b) that correspond to the Glut1–7 and Glut9–11 classes of Häring et al. (2018). In addition, we know that the GRPR cells receive excitatory synapses from each other (Polgár et al., 2023), as well as from a population of excitatory interneurons that are captured in the GRP:Cre line from the GENSAT project (Gong et al., 2003; Pagani et al., 2019), and which may correspond to the Glut8 population of Häring (Bell et al., 2020). GRP itself is widely expressed in the Glut5–12 populations (Häring et al., 2018), with the precursor protein pro-GRP being seen particularly in boutons belonging to GRP-GFP cells and cells that express NPFF (Gutierrez-Mecinas et al., 2023). As expected, boutons containing pro-GRP were commonly found presynaptic to the GRPR cells. Taken together, these findings suggest that a high proportion of excitatory synaptic input to the GRPR cells originates from excitatory interneurons, with relatively little coming from primary afferents. In addition, they show that most populations of excitatory interneurons in the SDH are presynaptic to GRPR cells. Because of the way in which our analysis was performed, we are not able to say whether individual GRPR cells receive direct synaptic input from both primary afferents and excitatory interneurons. However, because of the rarity of synapses from each of the classes of primary afferent that we analysed, it is very unlikely that any GRPR cells are exclusively innervated by primary afferents. We have previously reported that 7 out of 10 GRPR cells showed an increase in mEPSC frequency in the presence of capsaicin (Polgár et al., 2023), suggesting that most cells receive monosynaptic input from TRPV1-expressing primary afferents. It is therefore likely that individual GRPR cells receive synaptic input from both primary afferents and excitatory interneurons.

There are considerable differences in the pattern of excitatory synaptic input to different types of excitatory interneuron in the dorsal horn. For example, the GRP-GFP neurons (transient central cells) receive most of their excitatory synapses from primary afferents, while the substance P-expressing interneurons (radial cells) are thought to receive approximately equal numbers of synapses from primary afferents and excitatory interneurons (Polgár et al., 2020). Boyle et al. (2019) proposed that vertical cells received direct input from A-LTMRs, and we had found numerous contacts from VGLUT1-immunoreactive boutons (including A-LTMRs) onto vertical cells in the rat (Yasaka et al., 2014). This contrasts with our present finding that synapses from VGLUT1 boutons account for only a small proportion of the input to the GRPR cells. We recently identified another population of vertical cells, defined by the presence of pro-NPFF (Quillet et al., 2023), and it is possible that these cells receive a greater direct input from A-LTMRs.

Our findings are consistent with the suggestion that GRPR cells act as “tertiary pruritoceptors,” receiving input mainly from other excitatory interneurons, rather than primary afferents (Mishra and Hoon, 2013; Pagani et al., 2019). However, although, as predicted, the presynaptic neurons include those that express GRP, these only account for a minority of the input to the GRPR cells. The diversity of input from dorsal horn excitatory interneuron input to the GRPR cells, together with our previous finding that they are activated by noxious mechanical and thermal stimuli (Polgár et al., 2023), suggests that these neurons are involved in more somatosensory functions than just itch.

### Activation of projection neurons by GRPR cells

Most GRPR-expressing neurons in the dorsal horn are located in laminae I–IIo, and correspond to vertical cells (Polgár et al., 2023). It has long been assumed that ALS projection neurons in lamina I are a major postsynaptic target for vertical cells. This was originally suggested by Gobel (1978), who proposed, on the basis of Golgi studies, that axons derived from a class that he called stalked cells (and which largely correspond to vertical cells) arborised in lamina I and formed synapses on dendrites of projection neurons in this lamina. Subsequently, Lu and Perl (2005) identified excitatory synaptic connections from vertical cells to lamina I cells, some of which were retrogradely labelled from the thoracic spinal cord, and were therefore assumed to be projection cells. Cordero-Erausquin et al. (2009) injected a viral vector that results in retrograde trans-synaptic transport of GFP into the LPb and found that many vertical cells were labelled. They concluded that this reflected synaptic input from vertical cells to lamina I projection neurons.

However, Grudt and Perl (2002) noted that not all vertical cells have axons that enter lamina I, and observed that some sent axons into the dorsolateral fasciculus and/or the deep dorsal horn. We had shown that axons derived from GRPR cells arborised in the LSN and the deep dorsal horn, especially the lateral part of lamina V (Polgár et al., 2023), and here we provide evidence that these arbors originate (at least in part) from GRPR-expressing vertical cells in the SDH. The axonal projections of GRPR cells into lateral lamina V and the LSN are consistent with the relatively high level of Fos expression by ALS neurons in these regions when the GRPR cells were chemogenetically activated. Interestingly, the proportion of Fos-positive ALS cells in lamina I was much lower. This raises the possibility that projection cells in the deep dorsal horn and LSN receive a more powerful input from the GRPR cells than do lamina I ALS cells.

GRPR cells have mainly been implicated in itch (Sun and Chen, 2007; Sun et al., 2009), although we have shown that they are also activated by noxious stimuli and that chemogenetically exciting them in awake animals results in some pain-related behaviours (Polgár et al., 2023). Itch-responsive projection neurons are found in both lamina I and the deep dorsal horn, and these cells also respond to noxious stimuli (Davidson et al., 2007, 2012; Moser and Giesler, 2014). Pruritoceptive and nociceptive information is therefore likely to reach these neurons at least partly through GRPR cells in the SDH. However, LSN cells are thought to respond mainly to deep tissue input (Menetrey et al., 1980; Sikandar et al., 2017; Wercberger and Basbaum, 2019), and the projection of axons belonging to GRPR cells to this region may be more relevant to pain. Future studies will be needed to assess the extent to which GRPR-expressing vertical cells provide direct synaptic input to projection neurons in each of these regions.

## Data availability statement

The datasets presented in this study can be found in online repositories. The names of the repository/repositories and accession number(s) can be found below: http://dx.doi.org/10.5525/gla.researchdata.1500.

## Ethics statement

The animal study was approved by the University of Glasgow Animal Welfare and Ethical Review Board. The study was conducted in accordance with the local legislation and institutional requirements.

## Author contributions

RQ: Conceptualization, Formal analysis, Methodology, Writing – original draft, Writing – review and editing. MG–M: Conceptualization, Formal analysis, Methodology, Writing – review and editing. EP: Formal analysis, Methodology, Writing – review and editing. AD: Methodology, Writing – review and editing. KB: Conceptualization, Investigation, Methodology, Writing – review and editing. MW: Resources, Writing – review and editing. AT: Conceptualization, Funding acquisition, Investigation, Project administration, Supervision, Writing – original draft, Writing – review and editing.
