# Supplementary material for: Synaptic circuits involving gastrin-releasing peptide receptor-expressing neurons in the dorsal horn of the mouse spinal cord
Source: Front Mol Neurosci. 2023 Dec 7;16:1294994. doi: 10.3389/fnmol.2023.1294994 (PMC10742631; doi:10.3389/fnmol.2023.1294994)
Supplement: Supplementary file 1 [file Data_Sheet_1.docx]

Supplementary Material

Synaptic circuits involving gastrin-releasing peptide receptor-expressing neurons in the dorsal horn of the mouse spinal cord

Raphaelle Quillet^1†^, Maria Gutierrez-Mecinas^1†^, Erika Polgár^1^, Allen C Dickie^1^, Kieran A Boyle^1^, Masahiko Watanabe^2^, Andrew J Todd^1*^

*** Correspondence:**

Andrew Todd
andrew.todd@glasgow.ac.uk

## Supplementary Figures


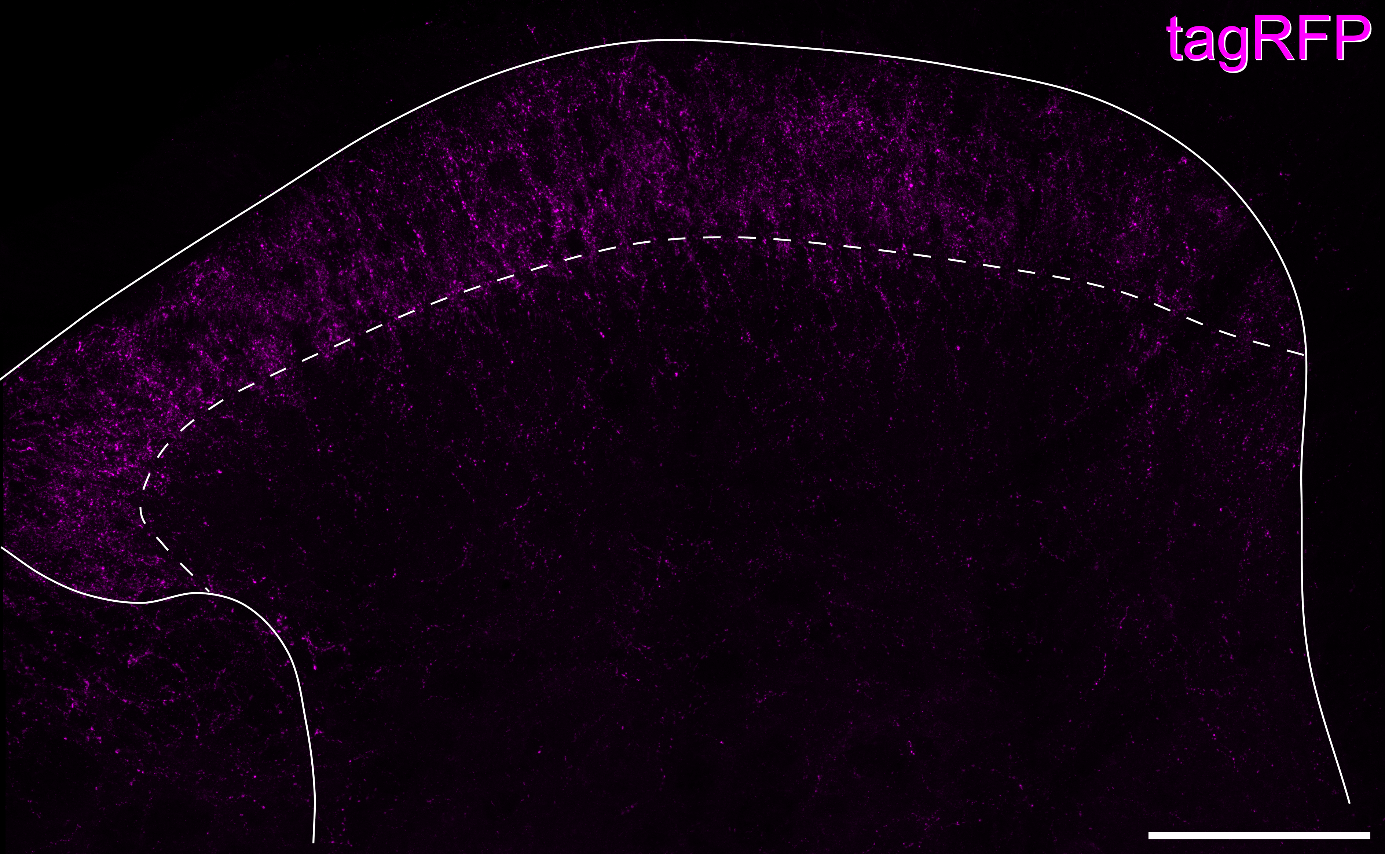
**Supplementary Figure 1.** Labelling for tagRFP in a GRPR^CreERT2^ mouse that received intraspinal injection of AAV.flex.PSD95-tagRFP. Note that tagRFP expression is largely restricted to the superficial dorsal horn, consistent with expression in GRPR-positive neurons. The image is a maximum intensity projection from 20 confocal optical sections at 0.5 μm z-separation. Scale bar = 100 μm.

**
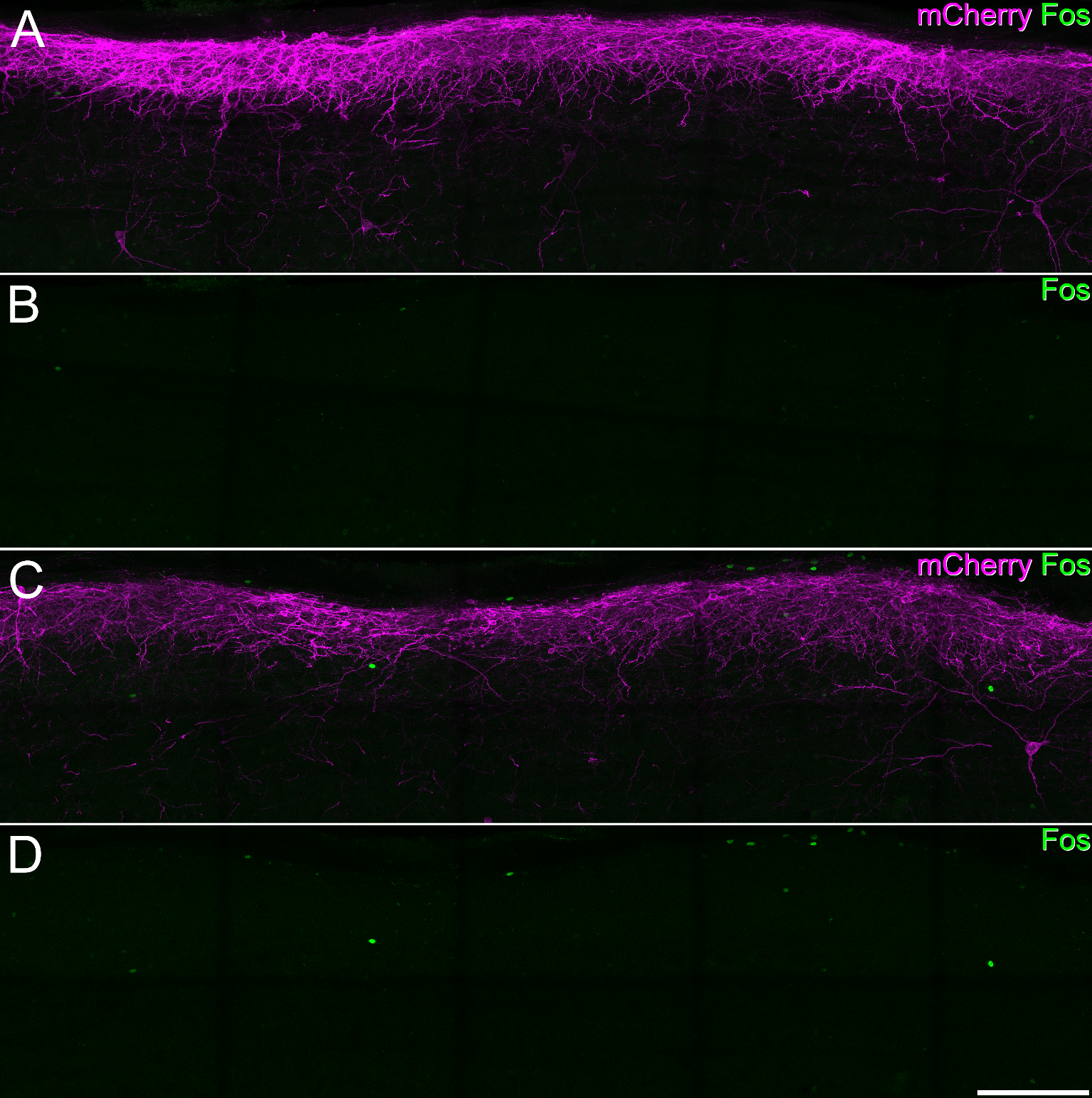
**

**Supplementary Figure 2.** Lack of Fos in GRPR^CreERT2^ mice that received intraspinal injection of AAV.flex.hM3Dq-mCherry and were treated with vehicle. **A**,**B** and **C**,**D** show sagittal sections through part of the lumbar spinal cord of both of the 2 vehicle-treated mice. Sections were immuno­stained for mCherry (magenta) and Fos (green). Although there are scattered Fos-positive cells, these are far less numerous than those seen after CNO treatment (compare with Figure 6). Images are maximum intensity projections through the full thickness of a 60 μm section. Scale bar = 200 μm.

**Supplementary Figure 3.**
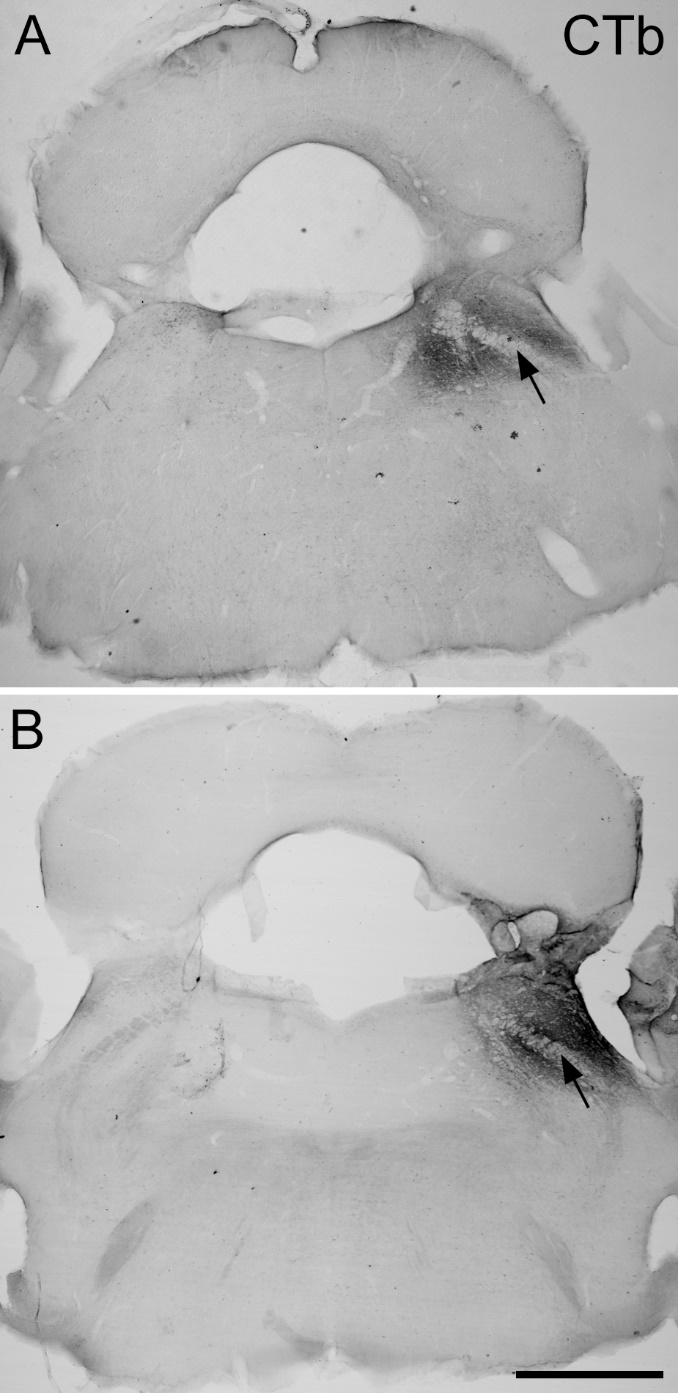
Cholera toxin B subunit (CTb) injection sites in the two mice that were used to investigate Fos upregulation in projection neurons following chemogenetic activation of GRPR cells. **A** is from the GRPR^CreERT2^ mouse and **B** is from the GRPR^Flp^;Phox2a::Cre;Ai32 mouse. The sections were reacted with an immunoperoxidase method to reveal CTb, which shows as the dark area. Arrows indicate the superior cerebellar peduncle. Scale bar = 1 mm.


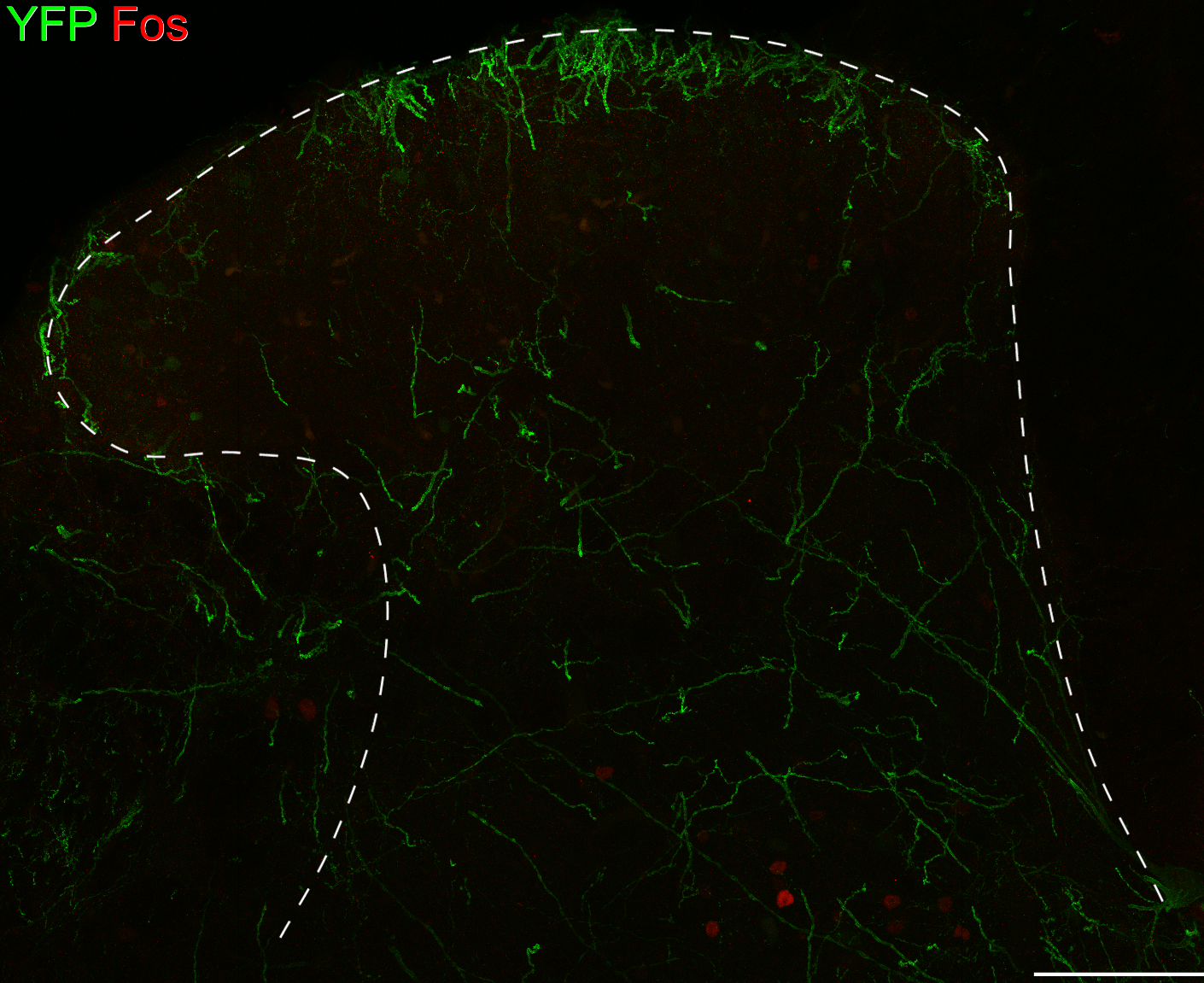


**Supplementary Figure 4.** Fos expression on the side contralateral to the intraspinal injection of AAV.frt.hM3Dq-mCherry in the GRPR^Flp^;Phox2a::Cre;Ai32 mouse that was used to investigate Fos upregulation in projection neurons following chemogenetic activation of GRPR cells. This image shows the contralateral side of the section illustrated in Fig 7. YFP labelling, corresponding to the Phox2a-positive cells is shown in green and Fos in red. Note that the fluorescence intensity levels are the same as those shown in Fig 7. Only a few cells with weak Fos labelling are visible on this side. Scale bar = 100 μm.


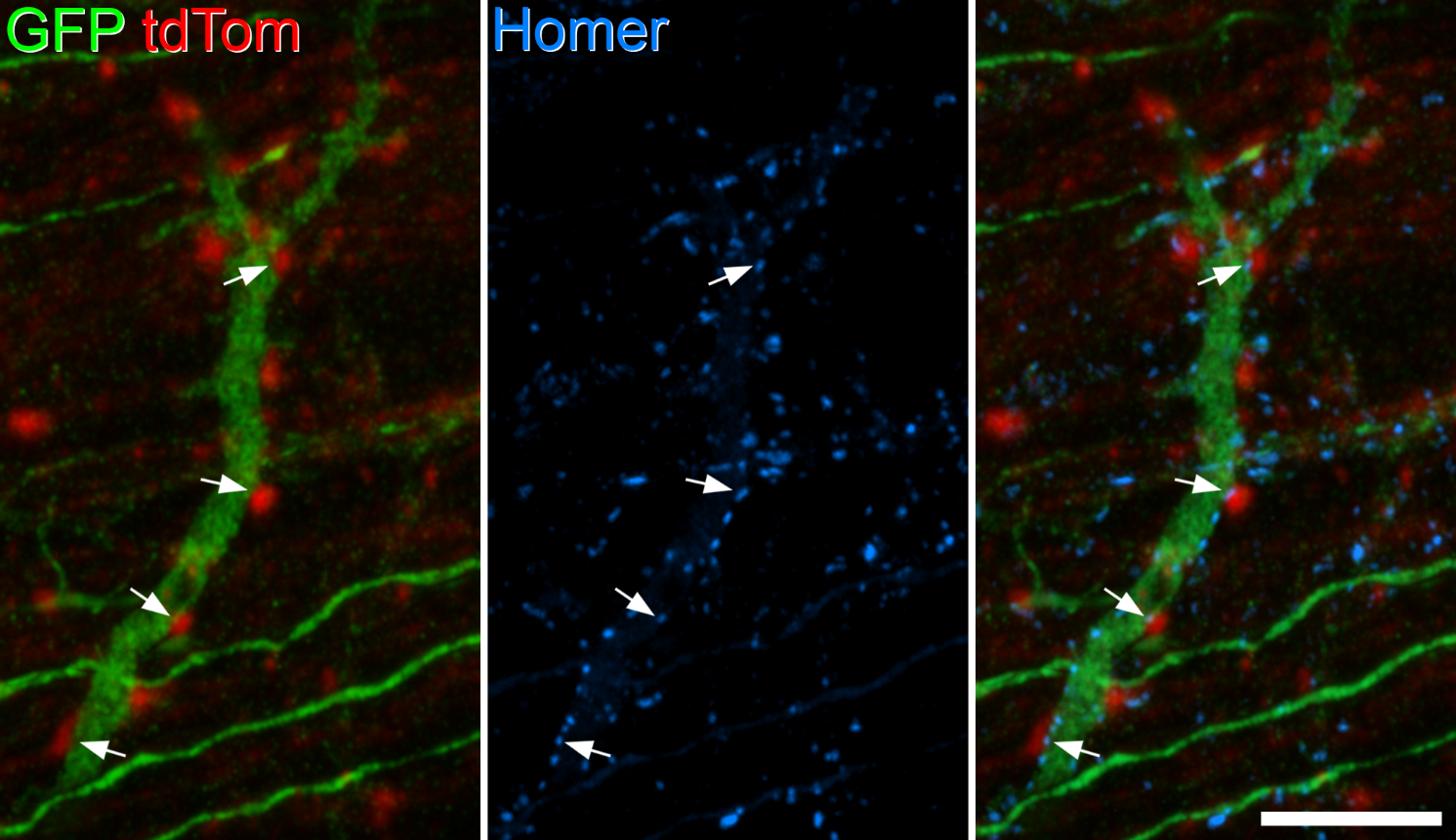


**Supplementary Figure 5.** Contacts from tdTomato-positive (tdTom) boutons and the dendrite of a GFP-labelled projection neuron seen in a horizontal section through the lateral reticulated part of lamina V from a GRPR^CreERT2^;Ai9 mouse that had received an injection of AAV.GFP into the contralateral lateral parabrachial area. The section has been immunostained to reveal the postsynaptic density protein Homer. A dendrite belonging to a projection neuron that is labelled with GFP is seen. Arrows point to 4 Homer puncta on the dendrite that are adjacent to tdTomato-positive boutons, presumably representing synapses from GRPR cells onto the dendrite of the projection neuron.

The image is a maximum intensity projection of 20 optical sections at 0.3 μm z-spacing. Scale bar = 10 μm.

**Supplementary Figure 6.** A GRPR cell that had undergone whole-cell patch clamp recording. Neurobiotin in the cell had been revealed with avidin-rhodamine. This cell, which has typical vertical cell morphology, gives rise to an axon that sends branches to both the lateral spinal nucleus (LSN) and the lateral part of lamina V (lateral V). The left arrow shows the point where the axon emerges from a dendrite, and the right arrow shows a branch point that gives rise to the ventrally-directed part of the axon
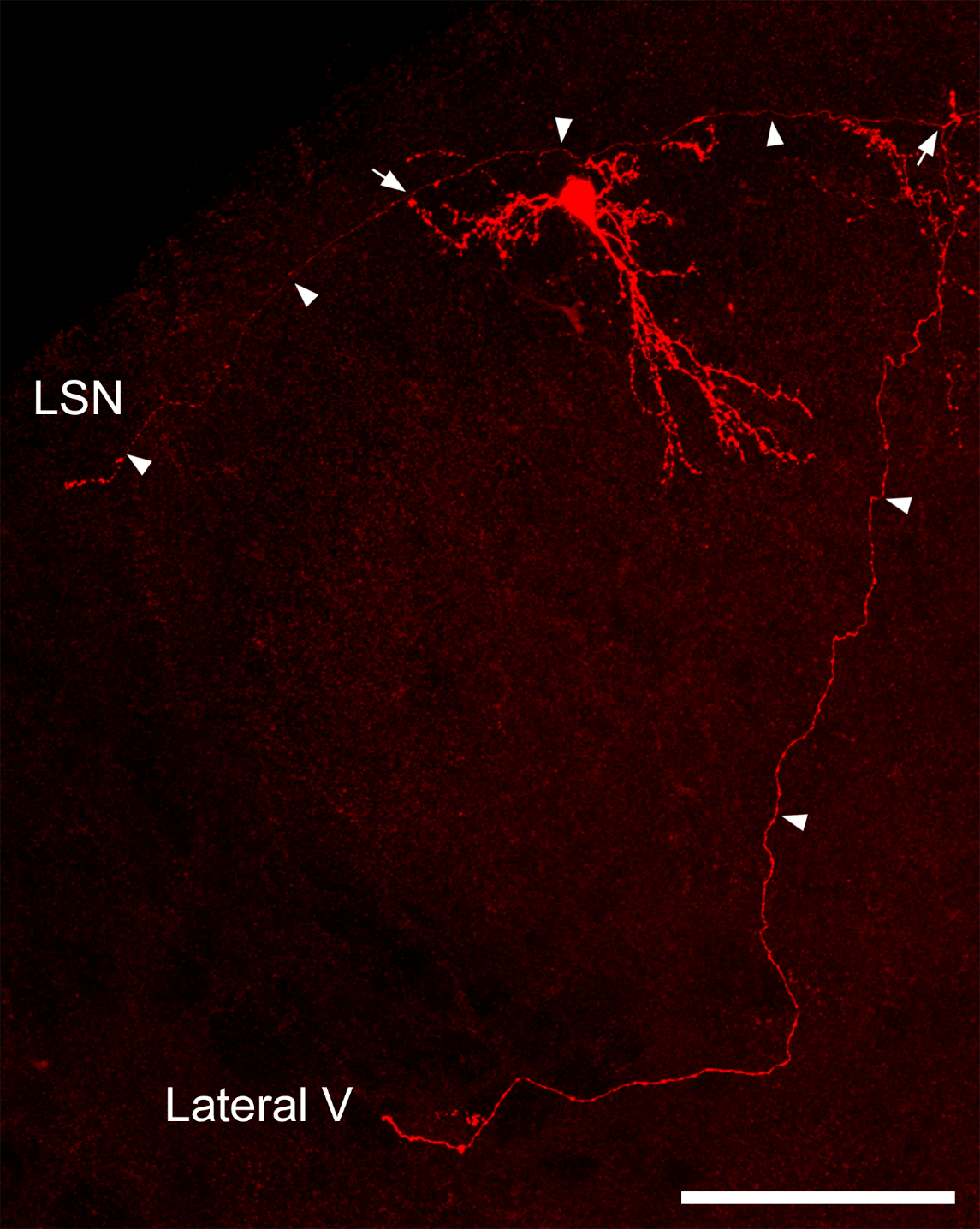
. The course of the axon is shown with arrowheads. Scale bar = 100 μm.
